# Nacre-mimic Reinforced Ag@reduced Graphene Oxide-Sodium Alginate Composite Film for Wound Healing

**DOI:** 10.1038/s41598-017-14191-5

**Published:** 2017-10-23

**Authors:** Xu Yan, Fei Li, Kang-Di Hu, Jingzhe Xue, Xiao-Feng Pan, Tao He, Liang Dong, Xiang-Ying Wang, Ya-Dong Wu, Yong-Hong Song, Wei-Ping Xu, Yang Lu

**Affiliations:** 1 Department of Pharmacy, Anhui Province Hospital, Hefei, Anhui 230001 P. R. China; 2 School of Pharmacy, Anhui University of Chinese Medicine, Hefei, Anhui 230012 P. R. China; 3 School of Chemistry and Chemical Engineering, School of Food Science and Engineering, Hefei University of Technology, Hefei, 230009 P. R. China; 4 College of Chemistry and Environmental Engineering, Shenzhen University, Shenzhen, Guangdong 518060 P. R. China; 5 Department of Chemistry, University of Science and Technology of China, Hefei, Anhui 230026 P. R. China

## Abstract

With the emerging of drug-resistant bacterial and fungal pathogens, there raise the interest of utilizing versatile antimicrobial biomaterials to treat the acute wound. Herein, we report the spraying mediated assembly of a bio-inspired Ag@reduced graphene-sodium alginate (AGSA) composite film for effective wound healing. The obtained film displayed lamellar microstructures similar to the typical “brick-and-mortar” structure in nacre. In this nacre-mimic structure, there are abundant interfacial interactions between nanosheets and polymeric matrix, leading to remarkable reinforcement. As a result, the tensile strength, toughness and Young’s modulus have been improved 2.8, 2.3 and 2.7 times compared with pure sodium alginate film, respectively. In the wound healing study, the AGSA film showed effective antimicrobial activities towards *Pseudomonas aeruginosa*, *Escherichia coli* and *Candida albicans*, demonstrating the ability of protecting wound from pathogenic microbial infections. Furthermore, *in vivo* experiments on rats suggested the effect of AGSA film in promoting the recovery of wound sites. According to MTT assays, heamolysis evaluation and *in vivo* toxicity assessment, the composite film could be applied as a bio-compatible material *in vitro* and *in vivo*. Results from this work indicated such AGSA film has promising performance for wound healing and suggested great potential for nacre-mimic biomaterials in tissue engineering applications.

## Introduction

Wounds on human body, as a ruptured skin and tissue, requires appropriate treatments to prevent infections and promote tissue regeneration^1^. In the past decades, a variety of dressing material have been developed for wound healing^2–4^. Among them, sodium alginate (SA) has received intensive interests as it was a natural, costless, and easily-obtained polysaccharide, showing good biocompatibility and the ability to facilitate wound healing by maintaining moist microenvironment^5,6^. However, the poor mechanical property and lack of function to withstand bacterial infection restrict the direct application of sodium alginate in wound healing^7^.

Inspired by the nature, introducing the inorganic nano-filler and fabricating “bricks-and-mortar” nacre-like microstructures became the most popular method for enhancing the mechanical properties of “soft” polymers in recent decade^8–12^. Graphene, one of the most popular 2D materials, has been increasingly studied as an ideal inorganic reinforcing constitutes due to their advantages over other materials in light weight, extraordinary mechanical properties and electrical conductivity, high flexibility and good ductility^13–16^. In addition, with high aspect ratio and superior processibility, graphene and its derivatives are also suitable candidates for growing and assembling functional components on their surface^17,18^. This unique characteristic could direct a feasible approach for fabricating functional graphene reinforced sodium alginates wound dressing materials.

Traditionally, organic antibiotics were encapsulated in wound dressing materials to combat with various microbial infections^5,19^. However, nowadays, the severe antimicrobial drug resistance has gradually weakened the actual anti-bacterial effect of organic medicines and threatens the public health^20^. As effective alternatives, metal or metal oxides inorganic nanoparticles (NPs) have been increasingly reported to kill microbial pathogens or inhibit their growth effectively^21–23^, thus providing a novel antimicrobial option, which could benefit the decreased usage of organic antibiotics, and in due reduce the emergence of resistance. Moreover, according to our previous work, Ag NPs decorated reduced graphene oxides (rGO) nanocomposites showed enhanced antibacterial effect and good bio-compatibility in skin treatment^24^. These positive results prompt us to further construct Ag NPs enriched rGO/sodium alginate nacre-mimic films with enhanced mechanical properties and anti-infection effect for wound healing.

In this paper, we constructed Ag@reduced graphene oxides-sodium alginate (AGSA) composites film with nacre-mimic microstructures which displayed enhanced mechanical performance comparing with pure SA film and Ag NPs@sodium alginate film. The AGSA film has effective antimicrobial activities against *Pseudomonas aeruginosa* (*P*. *aeruginosa*), *Escherichia coli* (*E*. *coli*) and *Candida albicans* (*C*. *albicans*). Further *in vivo* investigations showed such composite film prompted wound closure on rats. Moreover, the AGSA film was biocompatible to human endothelia cells and showed low haemolytic potential according to the MTT assay and heamolysis evaluation. We envisage that this AGSA film could find potential clinic translation in antimicrobial wound healing as a promising dressing materials.

## Results and Discussion

### The spraying assisted assembly of AGSA composite film

**Figure 1:**
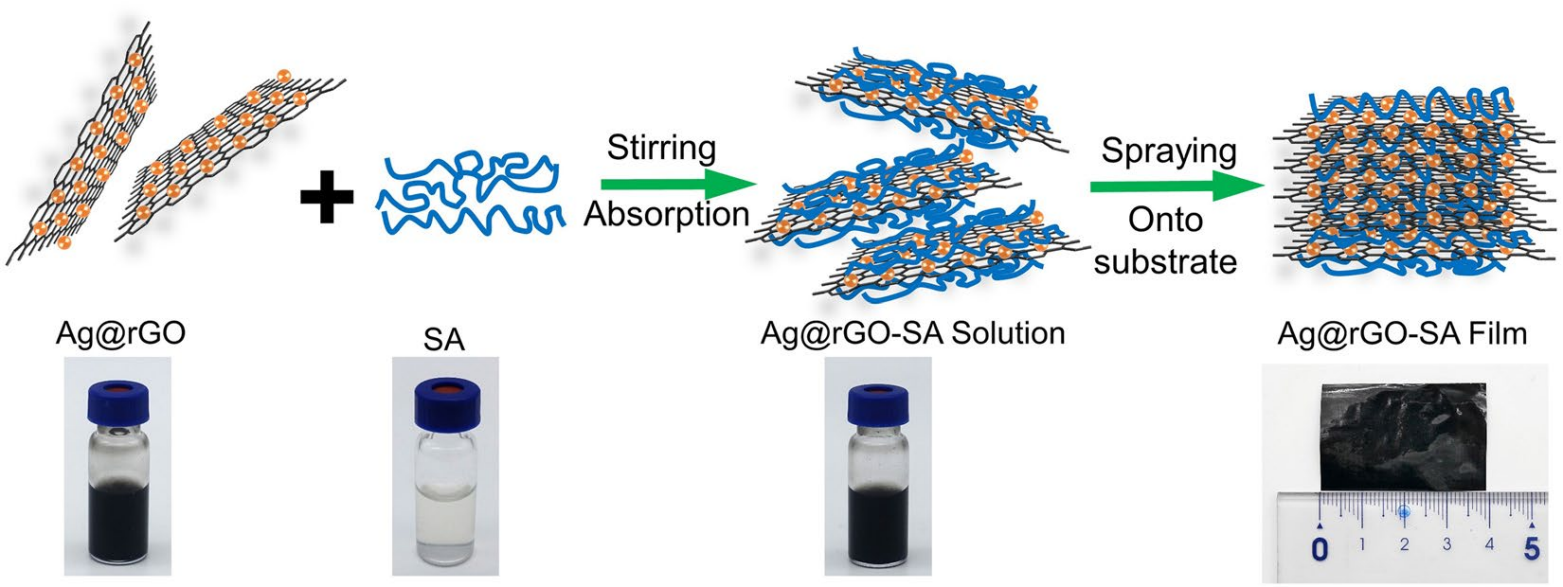
The processes for fabricating AGSA film.

Biomimetic synthesis is a fast growing and the most promising method for developing advanced materials^25^. The “brick-and-mortar” ordered microstructures, which endow nacre superior strength, stiffness, and toughness, have encourage us to reinforce SA film mechanical properties by filling inorganic building blocks and mimicking nacre-like microstructures. As illustrated in Fig. 1, the AGSA film was assembled via a simple process including the absorption of Ag@rGO sheets with SA molecules and spraying-assist assembly, and the content of Ag@rGO in this composite was 20% (w/w). Initially, Ag@rGO sheets were coated with SA molecules which could enable the formation of basic assembly building blocks. When the suspension of SA coated Ag@rGO sheets were sprayed onto a hot substrate, the ordered lamellar microstructures were formed after water-evaporation^26^. According to previous studies of alginate/graphene^27^ and Sodium alginate/montmorillonite bionanocomposites^28^, in the evaporation process of nano-platelets and alginate aqueous mixtures, the wet nanocomposites form a gel state, which push the nano-platelets to arrange layer-by layer. Moreover, in our manuscript, small drops of rGO-Ag and alginate mixture were sprayed continuously onto a hot plate, which accelerated the evaporation of water and the highly ordered arrangement of Ag@reduced graphene oxide-sodium alginate composite.

**Figure 2:**
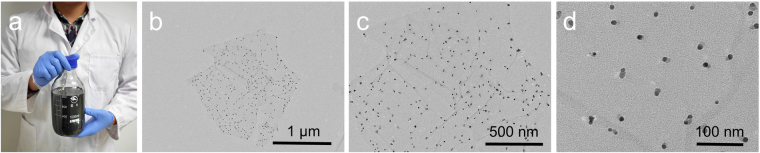
Preparation of Ag@rGO. (**a**) Mass production and (**b**–**d**) TEM images of the Ag@rGO sheets for assembling AGSA film.

**Figure 3:**
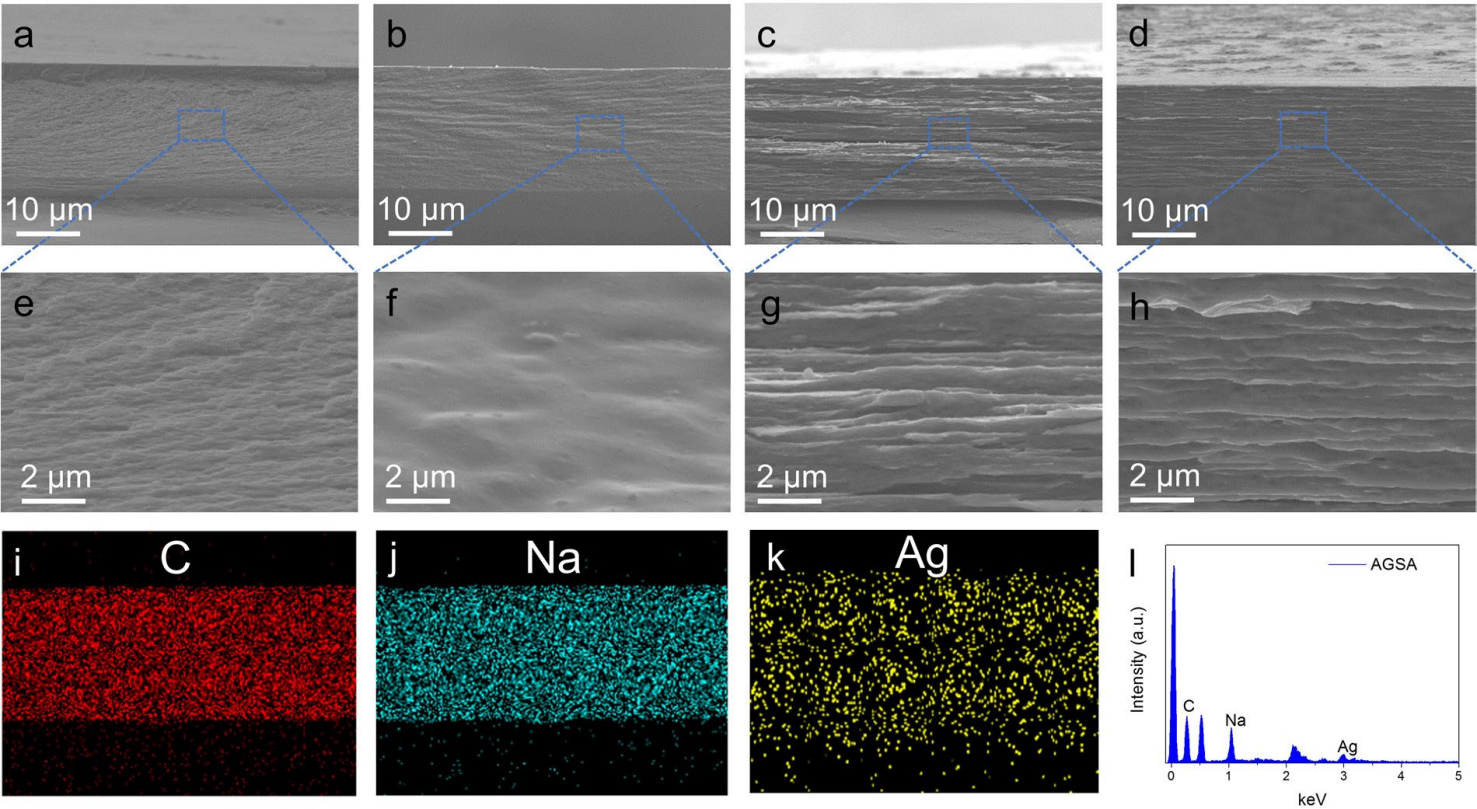
Cross-sectional SEM images of different samples: (**a**) SA film, (**b**) ASA film, (**c**) GSA film and (**d**) AGSA film; (**e**–**h**) High resolution images for (**a**–**d**), respectively; EDS analysis of cross-section of the AGSA film: (**i**–**k**) The corresponding full EDS mapping images of C, Na and Ag elements; (**l**) Spectrum of AGSA film.

**Figure 4:**
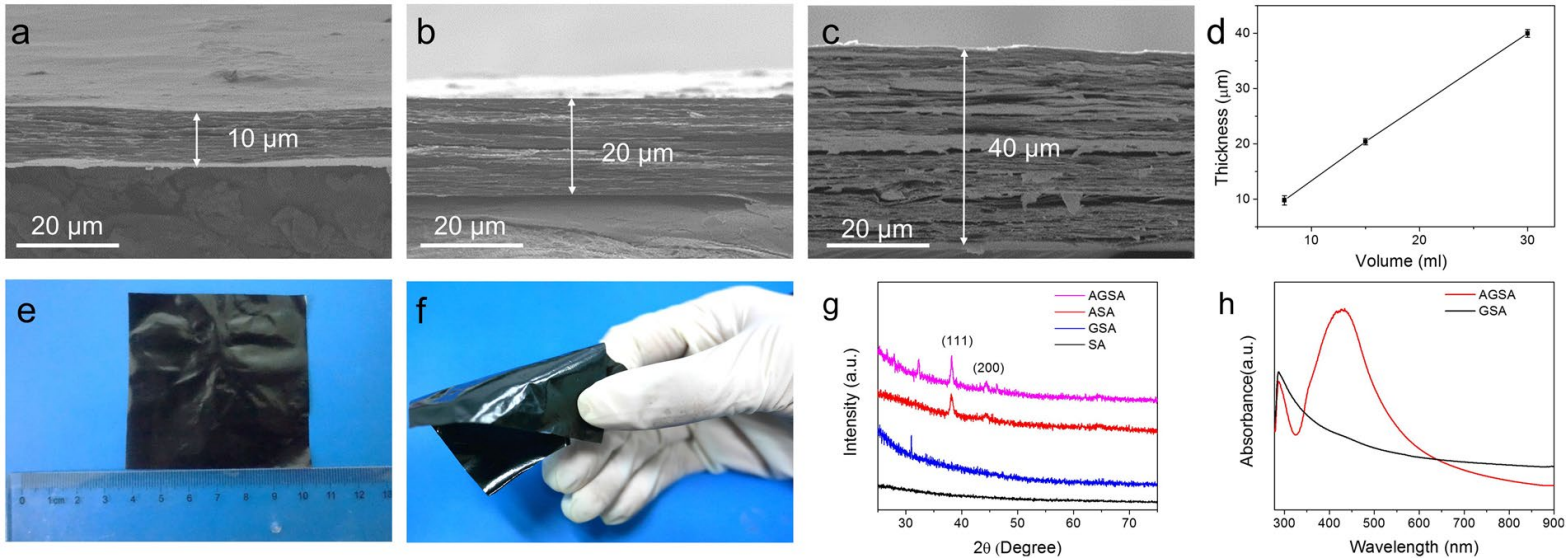
The thickness and dimension of AGSA film. (**a**–**c**) Cross-sectional SEM images for 10, 20, and 40 μm AGSA films, respectively; (**d**) Linear correlation between thickness of AGSA films and volume of solutions for spraying (error bars are standard deviation, n = 5); (**e**,**f**) Pictures of the AGSA film; (**g**) XRD and (**h**) UV-Vis spectrums of the samples.

Ag@rGO composites sheets were prepared according to a facile approach reported previously by our group^24^. Herein, as shown in Fig. 2a, mass production of this high quality composite nanosheets aqueous solution was successfully obtained at the concentration of 2 mg/mL, which could meet the demand of further fabrication of macroscale assemblies. Transmission electron microscopy (TEM) images confirmed that small Ag NPs were decorated uniformly on rGO sheets, with the size about 10 nm (Fig. 2b–d). The cross-section scanning electron microscopy (SEM) images of as-prepared films are presented in Fig. 3, and the thicknesses of these four samples (SA, ASA, GSA and AGSA films) were approximately 30 μm. Among them, SA and Ag-sodium alginate (ASA) films showed typical fracture morphology of polymers without lamellar microstructures (Fig. 3a,b,e,f). In contrast, ordered arrangement of rGO sheets can be clearly seen in graphene-sodium alginate (GSA) and AGSA films, demonstrating successful aligning and assembling of SA-coated rGO or Ag@rGO sheets during spraying and water-evaporation (Fig. 3c,d,g,h). Similar ordered microstructure have been reported in nacre-like film prepared from GO and various polymers^13,29^. EDS images and X-ray diffraction (XRD) analysis also confirmed the presence and well-distribution of Ag@rGO sheets in SA matrix (Fig. 3i–l). AGSA films with 10, 20 and 40 μm thick were also prepared respectively (Fig. 4a–c), indicating the thickness of as-prepared films increased with the increasing of spraying solution amount (Fig. 4d). From the digital pictures, as-prepared AGSA film was 6.5*6.5 cm in size, displaying a black appearance with slight metal luster (Fig. 4e), and the films were flexible to withstand bending (Fig. 4f). As shown in XRD patterns of ASA and AGSA films (Fig. 4g), the typical broadened diffraction peaks at 38.2° and 44.3° were indexed as the Ag (111) and (200)^24^, demonstrating the presence of small sized Ag NPs in such films. The absorption peaks around 400 nm arisen from surface plasmon resonance of colloidal silver further confirmed the presence of silver content in AGSA film (Fig. 4h). The typical transmission peaks of SA can be found in Fourier transform infrared spectroscopy (FTIR) patterns of SA, ASA, GSA and AGSA films (Supplementary Fig. 1), indicating SA serves as polymer matrix in all composite films. These results demonstrated the well-aligned AGSA composite film could be facilely fabricated by spraying method, and the thickness together with the dimension could be well controlled.

### Mechanical properties

**Figure 5:**
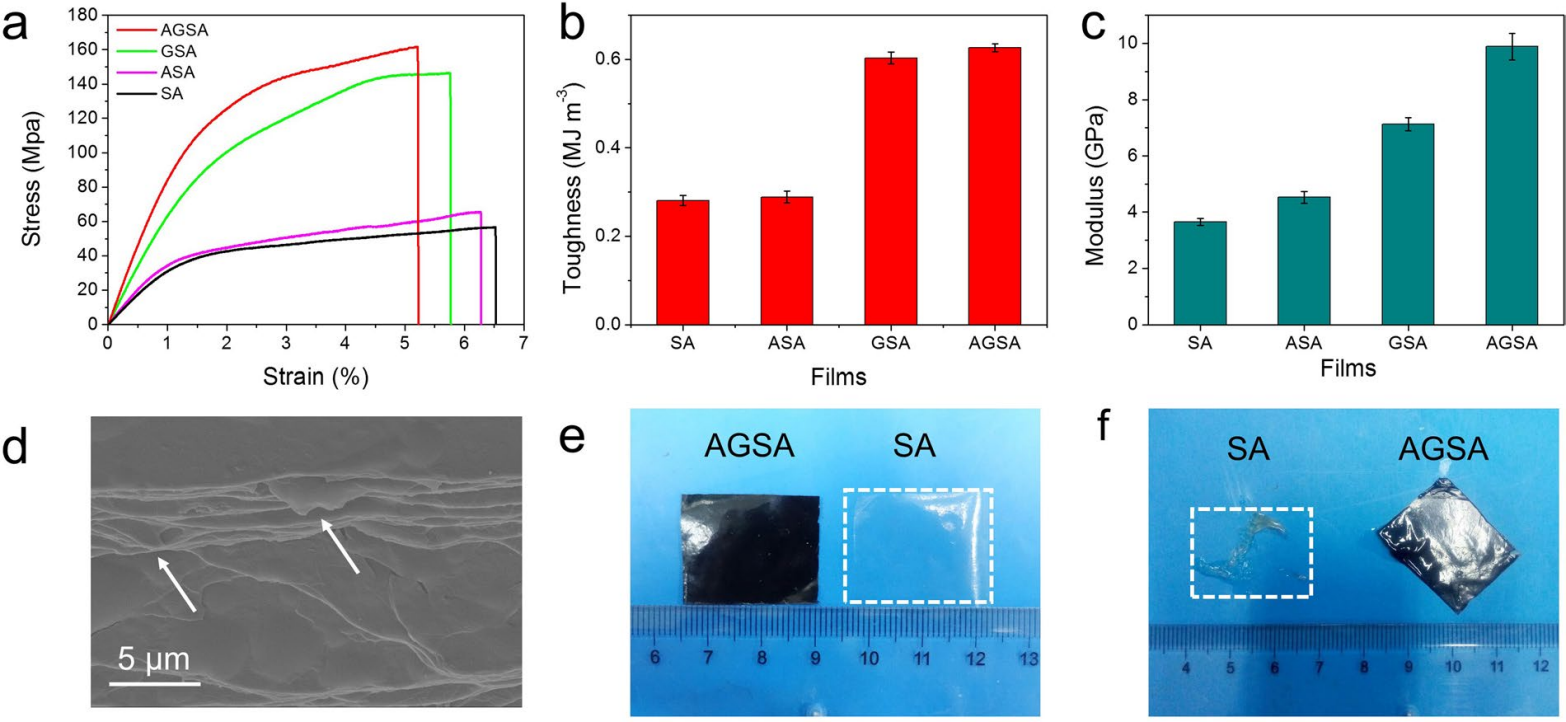
Mechanical test results of the as-prepared films. Stress-strain curves (**a**), Toughness (**b**) and Young’s modulus (**c**) of the different films; (**d**) Fractural morphology of AGSA film (White arrows refer to pulling out of the Ag@rGO sheets); (**e**) Pictures of AGSA and SA films before immersing in water; (**f**) Pictures of AGSA and SA films after immersing in water. Error bars are standard deviation, n ≥ 5.

The curing of wound sites may locate at motional parts of human body, and this requires wound healing materials having adequate mechanical stability. Dramatic enhancement in tensile strength was observed in typical stress–strain curves of the prepared films with inorganic fillings (Fig. 5a). It could be found that the tensile stress of AGSA film reached 161.2 ± 4.6 MPa, which was 2.8 times higher than pure SA film. ASA and GSA film were also tested as control experiments in tensile stress. After introducing Ag NPs in SA matrix, the tensile stress of ASA film slightly increased from 57.0 ± 5.0 MPa to 65.0 ± 4.9 MPa. Meanwhile, the incorporation of rGO sheets had a more profound effect as the tensile stress of GSA film reached 146 ± 6.0 MPa, which was nearly three times of pure SA film. Previous study suggested that the increase in strength and stiffness would sacrifice ductility or toughness^15^. However, in this study, there is only a slight difference in the tensile strain, which decreased from 6.5% of SA, to 6.3%, 5.7% and 5.2% for ASA, GSA and AGSA films respectively. This indicated the composite films still retained the ductility of polymeric matrix (Fig. 5a). Meanwhile, the toughness were enhanced with the increase in tensile stress: started from 0.28 ± 0.01 MJ m^−3^ of SA, and reached up to 0.29 ± 0.01, 0.60 ± 0.01 and 0.63 ± 0.01 MJ m^−3^ for ASA, GSA and AGSA films respectively (Fig. 5b). Moreover, the Young ‘s modulus of AGSA film was also 2.7, 2.2 and 1.4 times of those SA, ASA and GSA films, reaching up to 9.9 ± 0.5 GPa (Fig. 5c). Notably, the Young’s modulus was measured by the tensile testing in this study. Young’s modulus of these SA composites should be affected by analysis method, molecular weight of alginate as well as the sample humidity and film flatness, so the measurement parameters and conditions stayed the same for different films to avoid the measurement errors. In addition, the stabilities of SA and AGSA film were tested in moisture environment. After immersing in water for 30 secs, SA film collapsed and dissolved dramatically, while AGSA maintained its shape with partial swelling on the edges (Fig. 5e,f). The tensile stress-strain behaviors of SA and AGSA film were also investigated under wet condition. Generally, polymeric films, especially the SA films are highly sensitive to moisture, and their tensile stress should be reduced transparently in wet condition. The mechanical analysis was more complicated under wet condition than in dry state, as it is not easy to control the amount of the moisture and evaporation on the wet films as well as the clamping and gauge during the mechanical analysis. Although the humid values were hard to control in the mechanical measurement process, the tensile stress of AGSA film was still higher than that of pure SA film in wet condition, while the tensile strain of both films increased obviously (Supplementary Fig. 2). These findings suggested that the AGSA film could be a better choice in applying on humid wound, as the coverage could be more stable compared to SA film.

The significant improvement in mechanical properties of AGSA film was reasonable to take maximum benefit from the nacre-like lamellar assembly microstructures. Firstly, the ordered stacked microstructure enabled the efficient load transfer from the SA polymeric matrix to the nanoscale rGO sheets. As a well-known “hard” 2D material, graphene has outstanding tensile strength reaching about 130 GPa^10^, so rGO nanosheets could absorb more energy than SA matrix. As a result, the absorption and dissipation of the energy led to dramatic increase in tensile strength^30^. As shown in Fig. 5a, this explanation can be addressed as GSA film also displays obvious tensile strength than pure polymer film, which is in agreement with previous report^31^. Secondly, owing to the presence of Poly (sodium 4-styrenesulfonate) (PSS) and residue oxygen-containing groups on rGO sheets, hydrogen bonds may form between the hydroxyl groups of SA and oxygen atoms of PSS and rGO nanosheets. As a result, the interfacial interactions between SA matrix and rGO sheets were enhanced, and highly effective load transfer was prompted^10,32^. In Fig. 5d, the fracture morphology of AGSA film displays a typical nacre-mimic structure, and the limited pulling out of Ag@rGO sheets from the SA matrix provides evidence for increased interfacial interactions^33^. Moreover, as has been reported before, the presence of nanograins on aragonite platelets in nacre can play an important role in enhancing the mechanical properties^9,34^. In this case, the Ag NPs decorated on rGO sheets may act similarly to those nanograins. Through retarding the sliding of rGO sheets under tensile stress and introducing mechanically interlocking, Ag NPs contributed to the higher strength, which was in agreement with previous reports^35,36^. The role of Ag NPs can also be confirmed as AGSA film has higher tensile stress and modulus than GSA film (Fig. 5). With reinforced strength, toughness and Young’s modulus, this AGSA film was expected to have better mechanical stability during wound healing treatment.

### Antimicrobial activity

**Figure 6:**
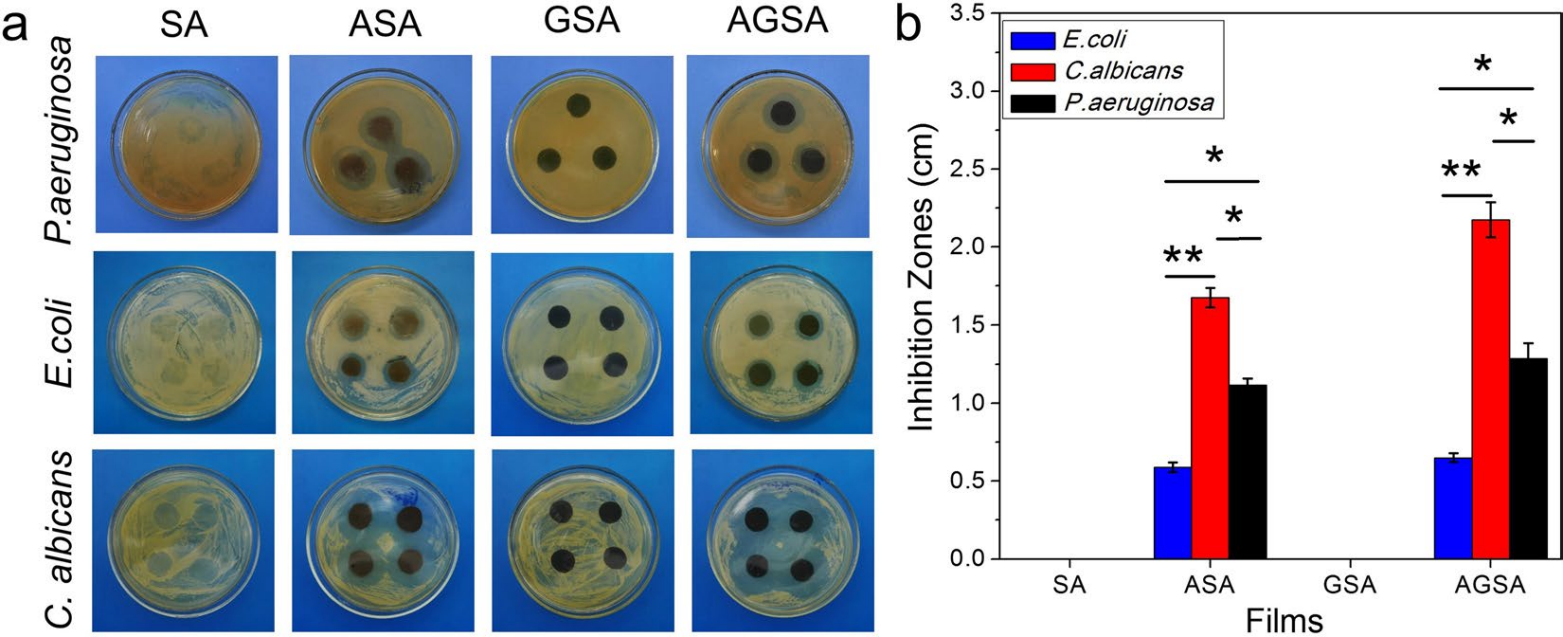
Results of antimicrobial investigations. (**a**) Pictures of the inhibition zones of different films; (**b**) Diameters of inhibition zones (Error bars are standard deviation; n = 3 for *P*. *aeruginosa*, n = 4 for *E*. *coli* and *C*. *albicans*; *p < 0.05, **p < 0.01).

The antimicrobial effects of as-prepared films were evaluated by investigating the inhibition zones. As shown in Fig. 6, inhibition zones around AGSA films were 1.30, 0.65 and 2.20 cm in diameter *P*. *aeruginosa*, *E*. *coli* and *C*. *albicans* respectively. These findings indicated that AGSA film exhibited effective protection capability against bacterial and fungal infection, especially towards *P*. *aeruginosa* which was the main concerned pathogen in wound. Pure SA, ASA and GSA films were examined to illustrate the origin of antimicrobial effect. Because the inhibition zones were absent in SA and GSA treatments but can be seen in ASA treatment (Fig. 6a), Ag NPs contributed majorly to the antimicrobial effect of AGSA. As previously reported, Ag NPs exhibited excellent inhibiting capability to microorganism^21,37^. Very recently, ultrafine Ag/AgCl nanoparticles coated on graphene was reported to inhibit microbial growth by generating oxidative species under visible-light irradiation, which can further promote healing in burn wound^38^. Herein, to illustrate the antimicrobial mechanism of Ag NPs in AGSA film, ICP analysis was conducted to investigate the leaching of Ag^+^ ions. According to Supplementary Fig. 3, the content of released silver increased with the monitoring time. After 7 days, 16.5% (w/w) of the total silver in AGSA film were released. This result was in consistent with previous conclusion regarding the role of released silver ions in antimicrobial effect of Ag NPs and Ag-based materials^39,40^. In our study, the antimicrobial effect of AGSA film could be attributed to the released silver ions, which could destroy cell membrane and disturb DNA replication^39^.

### *In vitro* and *in vivo* biological test

**Figure 7:**
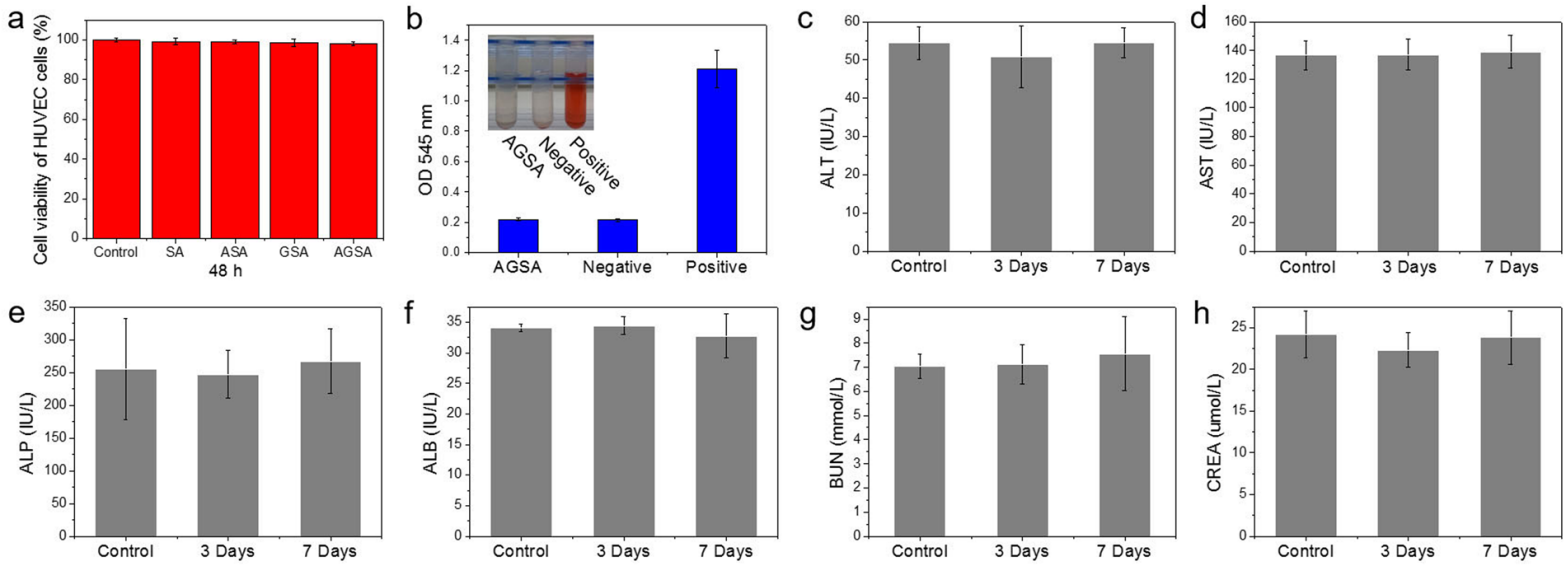
Biosafety evaluation of the AGSA film. (**a**) MTT assay results of the different films (n = 3); (**b**) Haemolysis evaluation of the AGSA film (n = 5); (**c**–**h**) Serum parameters of rats responding to functionality of liver and kidney. Error bars are standard deviation (n = 5).

The as-prepared AGSA film was composed of Ag NPs, reduced graphene oxide nanosheet and sodium alginate, and all components were biocompatible. In our previous report, no skin irritation on the epidermal laceration of rat was induced by Ag@rGO composite nanosheet^24^. In addition, water-soluble sodium alginate is a commercially used anionic natural polymer with broaden applications in biomedical area including drug delivery, tissue engineering and wound dressing^5^. First of all, the cytotoxicity of pure SA film and obtained ASA, GSA and AGSA composite films was studied using Human Umbilical Vein Endothelial Cells (HUVEC). After 2 days exposure to these films, no inhibition of cellular viability was detected in comparison with control group (untreated cells), confirming the good cyto-compatibility of these composite films including the AGSA film (Fig. 7a). In addition, the heamolysis evaluation of as-prepared AGSA film was performed using rat whole blood. In comparison with negative control (normal saline) and positive control (distilled water), the haemolytic potential was measured to be only 0.52% (Fig. 7b). According to previous report^41^, the haemolytic limit was considered to be 5%, which was 10 times higher than that of our AGSA film. As a result, our AGSA film with non cyto-toxicity and such a low haemolytic potential was compatible.

In order to assess whether the release of silver ions will cause adverse effects on hepatic and renal functions, the serum biochemistry tests were carried out. The blood of five rats was collected before the wound healing experiment to serve as normal samples. 3 and 7 days after wound-cutting and treatment with AGSA dressing on the same five rats, serum samples were further collected and measured respectively. The major serum markers of liver function including aspartate transaminase (ALT), alanine transaminase (AST), albumin (ALB) and alkaline phosphatase (ALP) were evaluated, and major indexes of kidney function including creatinine (CREA) and blood urea nitrogen (BUN) were measured in the meantime. All of these measured serum parameters stayed within normal ranges and revealed no obvious liver or kidney injury compared to the conditions without skin damage (Fig. 7c–h). As a result, our AGSA film was confirmed to be cyto- and haemo-compatible, and no disturbance of hepatic or renal function was induced after exposure to this film.

### *In vivo* Wound healing

**Figure 8:**
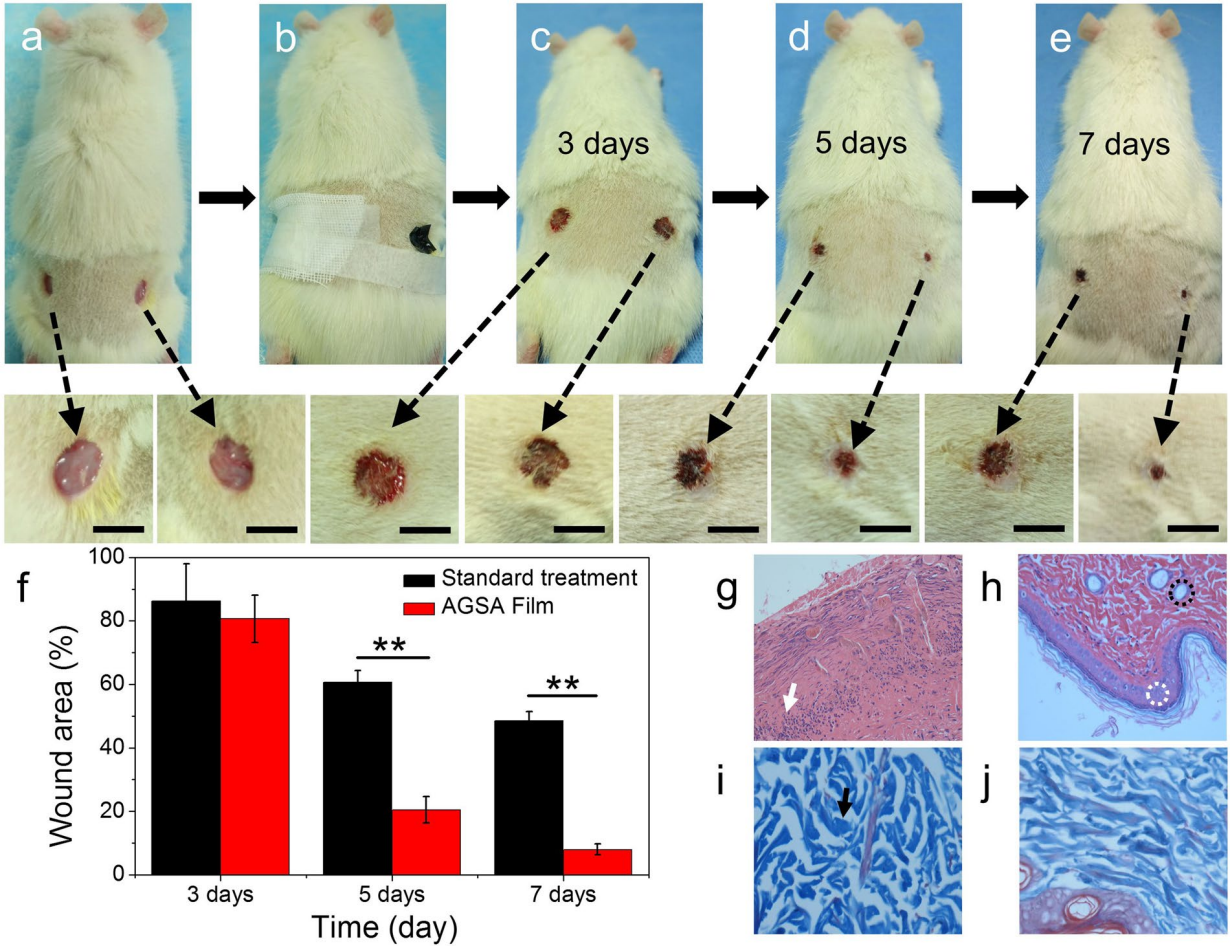
Observation of wound healing on rat (n = 5). (**a**) Two round acute wounds were cut at each side of depilated back skin of hip; (**b**) The wound of right side was covered with AGSA film in each rat, and the wound of left side was treated in standard disinfection treatment and coved by sterile gauze; (**c**–**e**) Pictures for wound healing after 3, 5 and 7 days respectively (bars = 5 mm); (**f**) Wound areas of standard and AGSA film treatment (Error bars are standard deviation); Photomicrographs showing section of skin tissues with H&E staining of wound sites with (**g**) standard and (h) AGSA film treatment after 7 days; Photomicrographs showing section of skin tissues with Masson’s Trichrome staining of wound sites with (**i**) standard and (**j**) AGSA film treatment after 7 days (40×). Where white arrow, black arrow, black dashed circle, white dashed circle indicated inflammatory cells, collagen fibers, gland and epidermis, respectively.

Alginate dressing has been widely used for the treatment of acute surgical wounds, for example, alginate matrix could load with stromal cell-derived factor-1 and release them continuously to accelerate wound closure rates and reduce scar formation^42^. Nowadays, various alginate dressings have been developed commercially, including Tegagen^TM^ (3 M Healthcare), Comfeel Plus^TM^ (Coloplast) and Kaltostat^TM^ (ConvaTec)^5^. Herein, five rats were employed to study the wound healing efficiency of our nacre-mimic reinforced Ag@rGO-sodium alginate composite film. After disinfection of skin using 75% ethyl alcohol and iodophor disinfectant, two round skin wounds with the diameter of about 0.5 cm were cut at each side of depilated back skin of hip. AGSA film was cut into proper size to cover the wound of right side in each rat. The wounds of left side were disinfected by rubbing alcohol and iodophor, and then covered with a sterile cloth as standard treatment control. In comparison with standard treatment control, significant wound shrunk and small scar could be observed in AGSA film treated group, without induction of inflammation (Fig. 8a–e). The size of wound area was measured by vernier caliper at 3, 5 and 7 days after the wound-cutting. As shown in Fig. 8f, the wound in standard treatment control group cured for 13.8%, 39.3% and 51.5% in area after 3, 5 and 7 days, respectively. Compared with the standard treatment control group, the wound area of AGSA film treatment group decreased quickly by 19.4%, 79.5% to 92% after 3, 5 and 7 days respectively, and new epidermis was regenerated prominently. In addition, as shown in Supplementary Fig. 4, when the wounds were remained without disinfection and dressing treatment, the control group showed much slow wound healing, and swelling and inflammation in wound appeared obviously.

The wound healing progress was further evaluated by histology analysis and Hematoxylin & Eosin and Masson’s Trichrome staining. After 7 days, a large number of inflammatory cells could be observed from the wound of standard treatment control (Fig. 8g), while some collagen fibers and glandular cavity appeared on the wound treated with AGSA dressing (Fig. 8h). Moreover, Masson’s Trichrome staining was used to examined collagen fibers formation in the wounds. As shown in the Fig. 8I,j, wound in standard treatment control group had a loose reticular arrangement of collagen fibers, and the space between the collagen was relatively large. In comparison, collagen bundles were relatively compact and showed a better ordered arrangement in the AGSA dressing treated wounds. These results indicated as-prepared AGSA film obviously accelerated effect on wound healing over a short period of time, and this composite film showed a great potential for further clinical translation.

## Conclusion

In summary, we demonstrated the assembly of AGSA composites film with nacre-like lamellar microstructures aiming at improving the performance of SA materials in wound healing. The as-prepared AGSA film showed enhanced mechanical properties compared with pure SA film. Its tensile strength, modulus and toughness were 2.8, 2.3 and 2.7 times of pure SA film, reaching up to 161.2 ± 4.6 MPa, 0.63 ± 0.01 MJ m^−3^ and 9.9 ± 0.5 GPa respectively. Meanwhile, AGSA film exhibited effective antimicrobial activities against both bacterial (*P*. *aeruginosa*, *E*. *coli*) and fungal pathogens (*C*. *albicans*). Importantly, MTT assay, heamolysis evaluation and *in vivo* toxicity assessment results suggested the promising safety for further medical applications. Furthermore, *in vivo* experiment on rat confirmed the effect of AGSA film in promoting the recovery of skin wound. These achievements, clearly demonstrated that our AGSA film possessed better mechanical stability and anti-infection capability than SA film, meanwhile maintained the safety *in vivo*. It is expected that AGSA film would have outstanding performance for wound healing and suggested a great potential for nacre-mimic biomaterials in tissue engineering applications.

## Methods

### Chemicals

Sodium alginate was purchased from Sangon Biotech Co. Ltd (Shanghai, China). Graphite powder, sulfuric acid (H_2_SO_4_), potassium permanganate (KMnO_4_), phosphorus pentoxide (P_2_O_5_), potassium peroxydisulfate (K_2_S_2_O_8_), hydrogen peroxide (H_2_O_2_), hydrochloric acid (HCl), and silver nitrate (AgNO_3_) were obtained from Sinopharm Chemical Reagent Co. Ltd (Shanghai, China). Poly (sodium 4-styrenesulfonate) (PSS), (Mw = 70000) was purchased from Sigma-Aldrich.

### Preparing of Ag NPs, rGO and Ag@rGO nanocomposites

Ag NPs were prepared according to Micro-wave assisted method reported by Hu *et al* ^43^. Graphene oxide nanosheets (GO) were synthesized from a modified Hammer’s method^44^. Ag@rGO composites were prepared according to our previous method^24^. Briefly, GO were firstly reduced to rGO using hydrazine hydrate as reductant. Then rGO-PSS solution were prepared and kept at 60 °C. AgNO_3_ solution was added into the rGO-PSS solution slowly with the assistance of double-jet pump at a speed of 0.5 ml h^−1^. Finally, Ag@rGO nanocomposites solution (1.5 mg/mL) were obtained after washing and diluting with deionized water.

### Assembly of nacre-like Ag@rGO-sodium alginate composites film

Spray assembly was employed to fabricate AGSA composites film. In a typical process for assembling AGSA film with, the Ag@rGO nanocomposites water solution (pH~6.5, 1.5 mg/mL) was mixed with sodium alginate water solution (pH~6.5, 2 mg/mL) under vigorous stirring for 10 min, and then subjected to sonication for 10 min in order to completely disperse Ag@rGO. Then, the mixture was sprayed onto heated glass substrate (140 °C). Finally, films were peeled off from substrate after solvent evaporated. The proportion of Ag@rGO in the film was fixed at 20%. To confirm the controllability of AGSA film, 7.5, 15 and 30 mL Ag@rGO-sodium alginate mixture solutions with the same compound proportion were sprayed into films with different thickness respectively. In addition, pure SA film, GSA film and ASA film were also prepared by similar spraying assisted process. The typical thicknesses of these composite films (SA, ASA, GSA and AGSA films) were about 30 μm.

### Characterization

Scanning electron microscopy (SEM, Zeiss Supra 40) and transmission electron microscopy (TEM, Hitachi HT7700) were used to investigate the morphology and structure of the samples. Energy dispersive spectroscopy (EDS, X-Max, Oxford) was used to analyze the constituent elements of the samples. X-ray diffraction (XRD) analysis was performed with a Philips X’Pert PRO SUPER X-ray diffractometer (Cu K α radiation). The mechanical properties of the as-prepared films were measured in tensile modern using Instron 5565 A testing machine at a load speed of 0.04 mm sec^−1^. The as-prepared films mechanical performance in wet condition were measured immediately after spraying water mist (~5 μL/s) onto the tested films for 3 s. The UV-visible (UV-Vis) spectrums of SA and AGSA films with the thickness of around 30 μm were measured by Shimadzu UV-2600 spectrometer. Inductive coupled plasma atomic emission spectrometer (ICP-AES, Perkin Elmer Optima 7300 DV) was used to determine the concentration of the released Ag^+^ ions. The mechanical properties of as-prepared films were measured using dual column electromechanical testing systems for tensile (Instron 5565 A equipped with 500 N load cells).

### Antimicrobial test

*P*. *aeruginosa* (ATCC 15692), *E*. *coli* (DH5α) and *C*. *albicans* (Sc 5314) were employed to evaluate the antimicrobial effect of the samples. Suspensions of *P*. *aeruginosa* and *E*. *coli* were obtained after culturing the two strains in Luria–Bertani (LB) medium for 20 h respectively. *C*. *albicans* was cultured in Yeast Extract Peptone Dextrose (YPD) medium for 20 h. All the microbial cells suspensions were diluted to 10^6^ CFU/mL, and each 100 μL such suspensions were spread onto LB or YPD agar plates uniformly. Then, film samples in round pieces with a diameter of 10 mm were attached onto such agar plates respectively. After incubation at 37 °C, 37 °C and 30 °C for 24 h for *P*. *aeruginosa*, *E*. *coli* and *C*. *albicans* respectively, antimicrobial effects of each sample were evaluated by measuring the diameters of inhibition zones.

### Detection of the released silver ions

In order to investigate the release of silver ions from AGSA film, the dialysis of AGSA film was carried out using a dialysis bag (MWCO = 1000 Da, Sangon Biotech, Shanghai, China). The filtrates were collected after 1, 3, and 7 days respectively, and ICP-AES was carried out to determine the amount of silver released from AGSA film.

### MTT assay

Human umbilical vein endothelial cell (HUVEC) was purchased from Anhui Medical University, which was employed to evaluate the cellular toxicity of films. Control group is normal cells without the exposure to any films, while cells in the other four groups were exposed to SA, ASA, GSA and AGSA films, respectively. After exposure to UV overnight, a series of films were cut into small pieces (2 mm *2 mm). HUVEC cells cultured in 96-wells plate were treated without or with these film pieces for 48 hours in DMEM medium (n = 3). Then these pieces were removed, and cells were washed with PBS and incubated with MTT for 4 h. The formazan crystals in each well were formed by the living cells, which was dissolved by DMSO. Finally, the absorbance at 490 nm was measured with Multiskan FC microplate photometer (Thermo, USA) to quantitatively calculate the cell viability.

### Animal experiments

According to previous procedure^41^, haemolytic potential of the final obtained AGSA film was evaluated using whole blood of rat with distilled water and PBS solution served as positive and negative control, respectively. Five SD rats (220 g bw, male) were anesthetized by chloral hydrate (10% in normal saline, 0.3 ml/100 g bw), and the depilated back skin of hip was chosed for the wound healing test. For the safety evaluation of AGSA film on five rats, blood samples were collected through the orbital venous plexus at different time points. These tests were carried out in accordance with the recommendations in the Guide for the Care and Use of Laboratory Animals of the National Institutes of Health. All protocols including heamolysis evaluation and wound healing experiment were reviewed and approved by the Institutional Animal Care and Use Committee (IACUC) of Anhui Medical University (LLSC20150134).

## Electronic supplementary material

Supplementary information

## References

[1] Parani M, Lokhande G, Singh A, Gaharwar AK (2016). Engineered nanomaterials for infection control and healing acute and chronic wounds. ACS Appl. Mater. Inter..

[2] Gil ES, Panilaitis B, Bellas E, Kaplan DL (2013). Functionalized silk biomaterials for wound healing. Adv. Healthc. Mater..

[3] Ong SY, Wu J, Moochhala SM, Tan MH, Lu J (2008). Development of a chitosan-based wound dressing with improved hemostatic and antimicrobial properties. Biomaterials.

[4] Castleberry SA (2016). Self-assembled wound dressings silence MMP-9 and improve diabetic wound healing *in vivo*. Adv. Mater..

[5] Lee KY, Mooney DJ (2012). Alginate: properties and biomedical applications. Prog. Polym. Sci..

[6] Silva JM, Caridade SG, Rui RC, Alves NM, Groth T (2015). pH responsiveness of multilayered films and membranes made of polysaccharides. Langmuir.

[7] Kim S, Moon JM, Choi JS, Cho WK, Kang SM (2016). Mussel-inspired approach to constructing robust multilayered alginate films for antibacterial applications. Adv. Funct. Mater..

[8] Yao HB, Ge J, Mao LB, Yan YX, Yu SH (2014). 25th anniversary article: artificial carbonate nanocrystals and layered structural nanocomposites inspired by nacre: synthesis, fabrication and applications. Adv. Mater..

[9] Mao LB (2016). Synthetic nacre by predesigned matrix-directed mineralization. Science.

[10] Zhang YY (2016). *Graphene-based art*ificial nacre nanocomposites. Chem. Soc. Rev..

[11] Wegst UGK, Bai H, Saiz E, Tomsia AP, Ritchie RO (2015). Bioinspired structural materials. Nat. Mater..

[12] Knöller A (2017). Strengthening of ceramic-based artificial nacre via synergistic interactions of 1D vanadium pentoxide and 2D graphene oxide building blocks. Sci. Rep..

[13] Cheng QF, Duan JL, Zhang Q, Jiang L (2015). Learning from nature: constructing integrated graphene-based artificial nacre. ACS Nano.

[14] Hu KS, Gupta MK, Kulkarni DD, Tsukruk VV (2013). Ultra-robust graphene oxide-silk fibroin nanocomposite membranes. Adv. Mater..

[15] Cui W (2014). A strong integrated strength and toughness artificial nacre based on dopamine cross-linked graphene oxide. ACS Nano.

[16] Qiu L (2014). Mechanically robust, electrically conductive and stimuli-responsive binary network hydrogels enabled by superelastic graphene aerogels. Adv. Mater..

[17] Huang X, Qi X, Boey F, Zhang H (2012). Graphene-based composites. Chem. Soc. Rev..

[18] Wang YF (2015). Graphene-directed supramolecular assembly of multifunctional polymer hydrogel membranes. Adv. Funct. Mater..

[19] Pritchard EM, Valentin T, Panilaitis B, Omenetto F, Kaplan DL (2013). Antibiotic-releasing silk biomaterials for infection prevention and treatment. Adv. Funct. Mater..

[20] Laxminarayan R, Sridhar D, Blaser M, Wang M, Woolhouse M (2016). Achieving global targets for antimicrobial resistance. Science.

[21] Zeng XK, McCarthy DT, Deletic A, Zhang XW (2015). Silver/reduced graphene oxide hydrogel as novel bactericidal filter for point-of-use water disinfection. Adv. Funct. Mater..

[22] Xue JZ (2014). A residue-free green synergistic antifungal nanotechnology for pesticide thiram by ZnO nanoparticles. Sci. Rep..

[23] Miller KP, Wang L, Benicewicz BC, Decho AW (2015). Inorganic nanoparticles engineered to attack bacteria. Chem. Soc. Rev..

[24] Xu WP (2011). Facile synthesis of silver@graphene oxide nanocomposites and their enhanced antibacterial properties. J. Mater. Chem..

[25] Zan GT, Wu QS (2016). Biomimetic and bioinspired synthesis of nanomaterials/nanostructures. Adv. Mater..

[26] Richardson JJ, Bjornmalm M, Caruso F (2015). Technology-driven layer-by-layer assembly of nanofilms. Science.

[27] Vilcinskas K (2015). Tunable order in alginate/graphene biopolymer nanocomposites. Macromolecules.

[28] Zlopasa J, Norder B, Koenders EAB, Picken SJ (2015). Origin of highly ordered sodium alginate/montmorillonite bionanocomposites. Macromolecules.

[29] Wan S (2015). Use of synergistic interactions to fabricate strong, tough, and conductive artificial nacre based on graphene oxide and chitosan. ACS Nano.

[30] Zhang J (2016). Multiscale deformations lead to high toughness and circularly polarized emission in helical nacre-like fibres. Nat. Commun..

[31] Chen K, Shi B, Yue YH, Qi JJ, Guo L (2015). Binary synergy strengthening and toughening of bio-inspired nacre-like graphene oxide/sodium alginate composite paper. ACS Nano.

[32] Podsiadlo P (2007). Ultrastrong and stiff layered polymer nanocomposites. Science.

[33] Wan S (2015). Synergistic toughening of graphene oxide-molybdenum disulfid-thermoplastic polyurethane ternary artificial nacre. ACS Nano.

[34] Wang L (2017). *Graphene-co*pper composite with micro-layered grains and ultrahigh strength. Sci. Rep..

[35] Xia S (2015). Nanoasperity: structure origin of nacre-inspired nanocomposites. ACS Nano.

[36] Bouville F (2014). Strong, tough and stiff bioinspired ceramics from brittle constituents. Nat. Mater..

[37] Cao F (2017). An efficient and benign antimicrobial depot based on silver-infused MoS_2_. ACS Nano.

[38] Zhou Y (2016). Biomedical potential of ultrafine Ag/AgCl nanoparticles coated on graphene with special reference to antimicrobial performances and burn wound healing. ACS Appl. Mater. Inter..

[39] Le Ouay B, Stellacci F (2015). Antibacterial activity of silver nanoparticles: a surface science insight. Nano Today.

[40] Xue JZ (2016). Integrated nanotechnology for synergism and degradation of fungicide SOPP using micro/nano-Ag_3_PO_4_. Inorg. Chem. Front..

[41] Xu YJ (2016). Lanthanide co-doped paramagnetic spindle-like mesocrystals for imaging and autophagy induction. Nanoscale.

[42] Rabbany SY, Pastore J, Yamamoto M, Miller T, Rafii S (2010). Continuous delivery of stromal cell-derived factor-1 from alginate scaffolds accelerates wound healing. Cell Transplant..

[43] Hu B, Wang SB, Wang K, Zhang M, Yu SH (2008). Microwave-assisted rapid facile “green” synthesis of uniform silver nanoparticles: self-assembly into multilayered films and their optical properties. J. Phys. Chem. C.

[44] Chen P (2014). Nitrogen-doped nanoporous carbon nanosheets derived from plant biomass: an efficient catalyst for oxygen reduction reaction. Energy Environ. Sci..

