# Supplementary material for: Nacre-mimic Reinforced Ag@reduced Graphene Oxide-Sodium Alginate Composite Film for Wound Healing
Source: Sci Rep. 2017 Oct 23;7:13851. doi: 10.1038/s41598-017-14191-5 (PMC5653744; doi:10.1038/s41598-017-14191-5)
Supplement: Supplementary file 1 — Supplementary information [file 41598_2017_14191_MOESM1_ESM.doc]

Supplementary information for

“Nacre-mimic Reinforced Ag@reduced Graphene Oxide-Sodium Alginate Composite Film for Wound Healing”

Xu Yan^1,#^, Fei Li^2,#^, Kang-Di Hu^2,#^, Jingzhe Xue^3,4,*^, Xiao-Feng Pan^2^, Tao He^2^, Liang Dong^4^, Xiang-Ying Wang^2^, Ya-Dong Wu^2^, Yong-Hong Song^2^, Wei-Ping Xu^1,5,*^ & Yang Lu,^2,*^

^1^School of Pharmacy, Anhui University of Chinese Medicine, Hefei, Anhui 230012, P. R. China.

^2^School of Chemistry and Chemical Engineering, School of Food Science and Engineering, Hefei University of Technology, Hefei, Anhui 230009, P. R. China.

^3^College of Chemistry and Environmental Engineering, Shenzhen University, Shenzhen, Guangdong, 518060, P. R. China.

^4^Department of Chemistry, University of Science and Technology of China, Hefei, Anhui 230026, P. R. China

^5^Department of Pharmacy, Anhui Province Hospital, Hefei, Anhui 230001, P. R. China

^#^These authors contributed equally to this work.

^*^Corresponding authors: J. Xue. (email: jzxue@ustc.edu.cn), W.-P. Xu. (email: wpxu@mail.ustc.edu.cn) and Y. Lu. (email: yanglu@hfut.edu.cn)


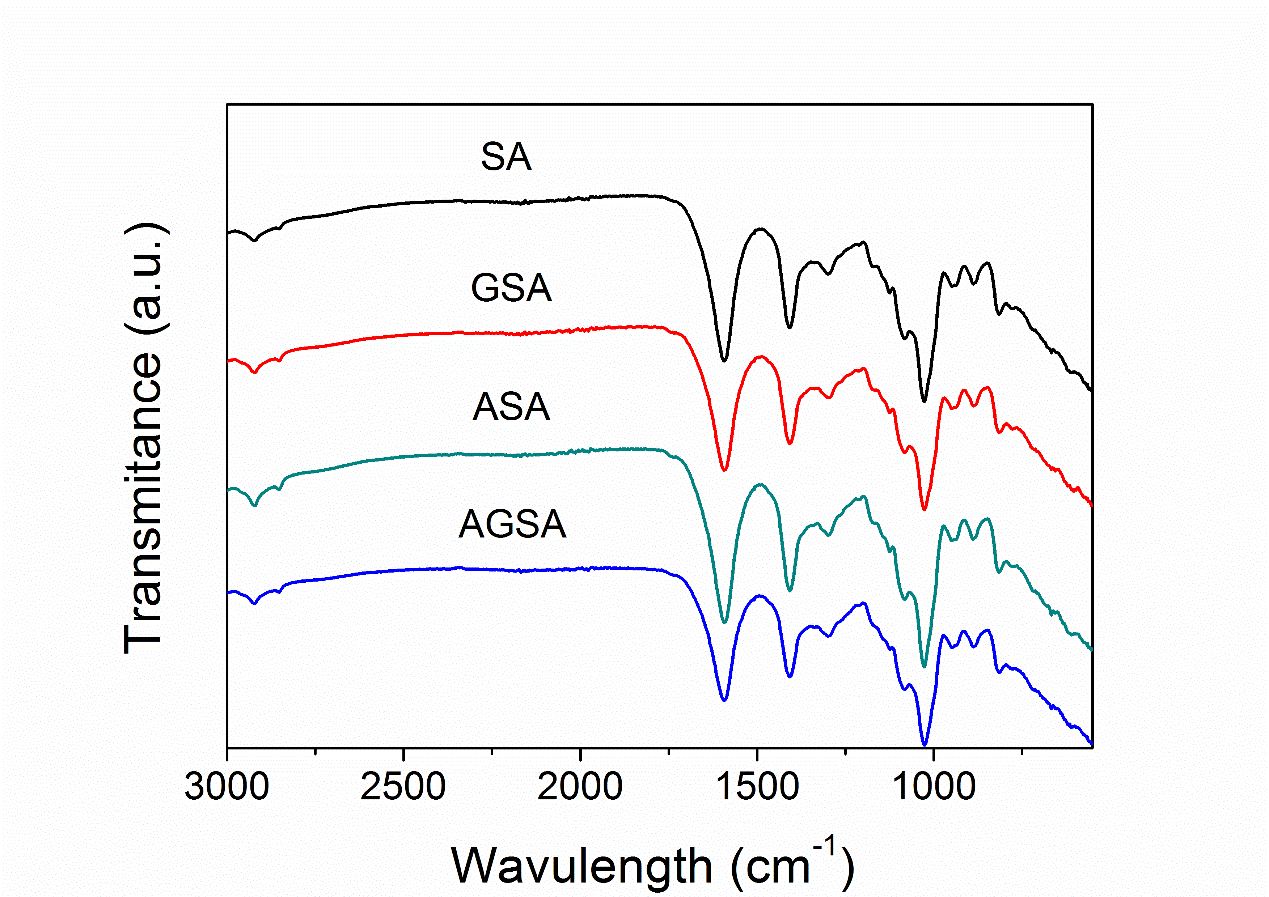


**Supplementary Figure 1. FTIR spectrum of the sample films.**


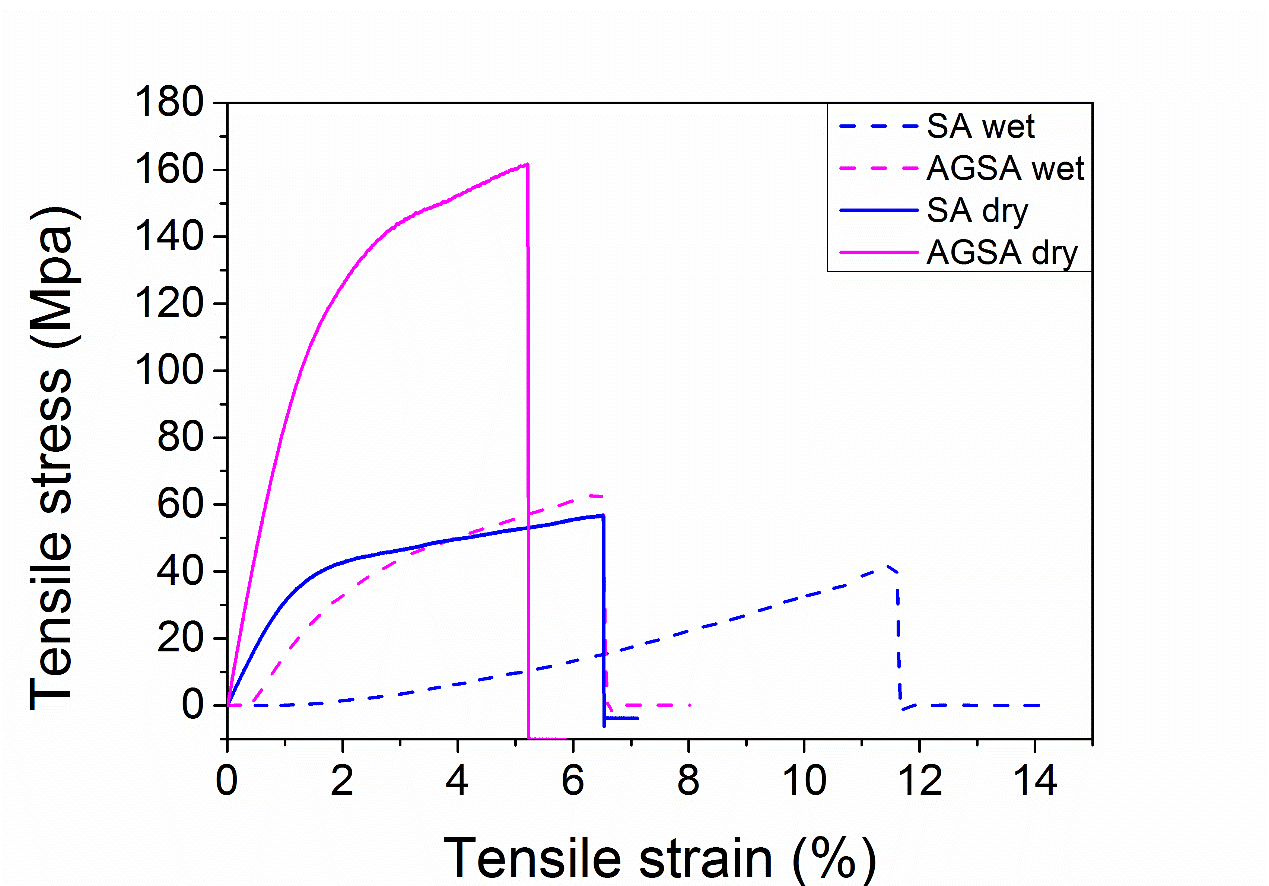


**Supplementary Figure 2.** **Mechanical performance of SA and AGSA film under wet condition**.


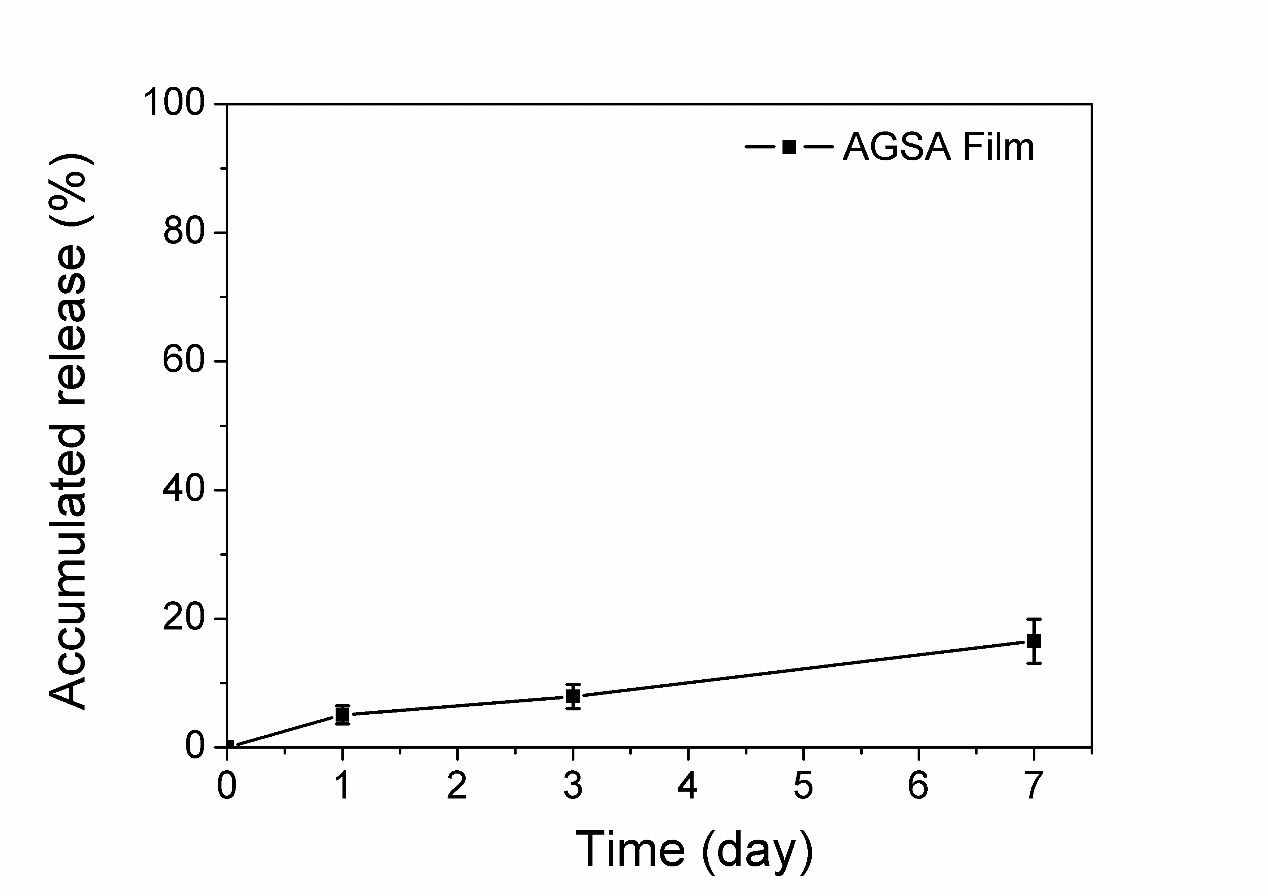


**Supplementary Figure 3. Accumulated silver ions released from AGSA film.** Error bars are standard deviation


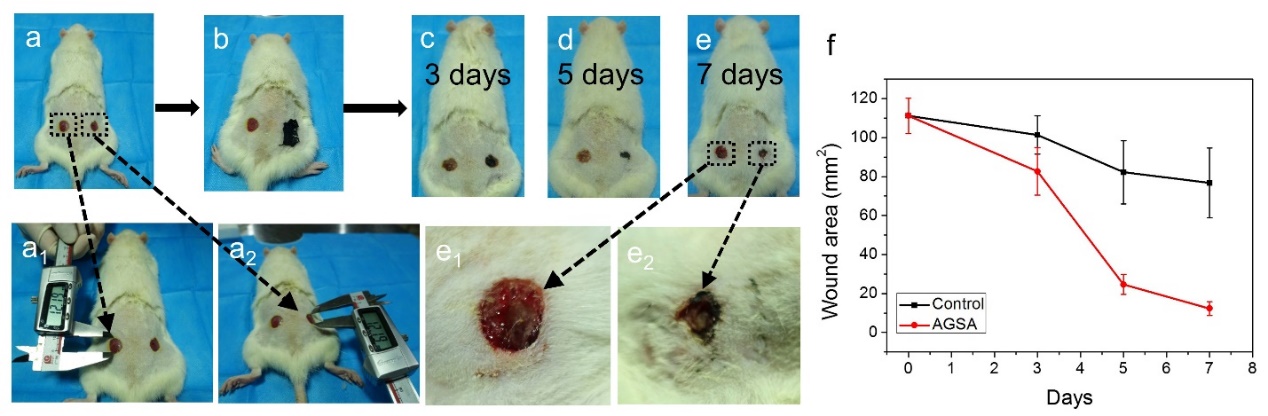


**Supplementary Figure 4. Observation of wound healing on rat (n=5).** (a) Two round acute wounds were cut at each side of depilated back skin of hip; (b) The wound of right side was covered with AGSA film in each rat, remaining the wound of left side without any treatment as control; (c-e) Pictures for wound healing after 3, 5 and 7 days respectively; (a1, a2) Diameters of wound sites; Visual observation of surface healing upon wound sites (e1) without treatment and (e2) treated with AGSA film after 7 days; (f) Wound area untreated control and treated with AGSA film (Error bars are standard deviation).
